# Lactate and lactylation: emerging roles in autoimmune diseases and metabolic reprogramming

**DOI:** 10.3389/fimmu.2025.1589853

**Published:** 2025-06-27

**Authors:** Wenjun Liu, Ruhui Yang, Yuxin Zhan, Xuanyu Yang, Haimin Zeng, Bofan Chen, Jiahao Zeng, Tianheng Hu, Jie Hu, Qi Xiao, Yinjin Shao, Xiang Chen

**Affiliations:** ^1^ Department of Rehabilitation Medicine, The Second Affiliated Hospital, Jiangxi Medical College, Nanchang University, Nanchang, China; ^2^ Queen Mary School, Jiangxi Medical College, Nanchang University, Nanchang, Jiangxi, China; ^3^ The Second Clinical Medical College, Jiangxi Medical College, Nanchang University, Nanchang, China; ^4^ The Ophthalmology and Optometry College, Jiangxi Medical College, Nanchang University, Nanchang, China; ^5^ Department of Pharmacy, Meijiang Town Shuidong Health Center, Ningdu, Jiangxi, China; ^6^ The First Clinical Medical College, Jiangxi Medical College, Nanchang University, Nanchang, China; ^7^ Department of Rehabilitation Medicine, Ganzhou People’s Hospital, Ganzhou, China

**Keywords:** lactate, lactylation, autoimmune diseases, metabolic reprogramming, epigenetics

## Abstract

Autoimmune diseases are a set of conditions in which the immune system incorrectly identifies and attacks the body’s own healthy tissue, severely compromising patient health. While current treatments can somewhat control disease progression, their long-term effectiveness remains limited, necessitating the development of more effective therapeutic approaches. Lactate and lactylation are critical links between metabolic reprogramming and epigenetics. As an emerging epigenetic modification, lactylation induced by lactate is closely associated with the onset of autoimmune diseases. Lactylation can be categorized into histone and nonhistone modifications, both of which play pivotal roles in cellular functions and pathophysiological processes through distinct regulatory mechanisms. Lactylation impacts immune cell function by regulating metabolic reprogramming and signaling pathways. In autoimmune diseases, immune cell metabolic reprogramming controls lactylation levels through metabolic byproducts, and lactylation, in turn, modulates the cellular metabolism by altering the transcription and structure of key enzymes. These interconnected processes collectively drive disease progression. To better understand the role of lactate and lactylation in the pathogenesis of autoimmune diseases, this review synthesizes the effects on specific immune cells, examining their dual effects on immune system function and their particular impacts on two common autoimmune diseases—rheumatoid arthritis (RA) and systemic lupus erythematosus (SLE). By combining the established role of lactate in immune metabolic reprogramming with the emerging understanding of the influence of lactate-induced lactylation on epigenetic regulation, this paper explores the relationship between lactylation and the progression of autoimmune diseases. This approach aims to enhance the understanding of the interplay between epigenetics and metabolism in autoimmune disease development, providing new perspectives for future therapeutic strategies. Studies collectively indicate that treatment can be improved through regulating key enzymes involved in lactylation, targeting lactate production pathways, integrating innovative approaches with current therapies, and adopting personalized treatment strategies.

## 1 Introduction

Autoimmune diseases occur when the immune system erroneously targets the body’s own healthy cells and tissues, leading to chronic inflammation, tissue damage, and organ dysfunction (1). Approximately 5% of the global population is affected by autoimmune diseases (2). RA and SLE are common autoimmune diseases. RA is driven by Th1 or Th17 proinflammatory T-cell responses and is mediated by various antibodies that target self-antigens, causing joint and bone destruction through inflammation-induced infiltration (3–5). SLE, a chronic systemic disease, is caused by autoantibodies (especially those targeting nucleic acids and nucleoproteins) and immune complexes formed by self-antigens (6), with most affected organs exhibiting inflammatory manifestations (7).

The emerging concept of “immunometabolism” suggests that immune cells undergo metabolic reprogramming upon activation and differentiation, a process through which cells adjust their metabolic pathways to adapt to environmental changes or meet specific functional requirements. Different immune cells exhibit distinct metabolic profiles, and fluctuations in metabolite concentrations can modulate the intensity of the immune response (8, 9). Metabolic reprogramming plays a crucial role in immune cell differentiation, proliferation, and effector functions. However, metabolite accumulation can disrupt immune homeostasis and trigger the onset of autoimmune diseases (10). The metabolic reprogramming of immune cells is closely associated with the development and progression of autoimmune diseases (11). Glucose serves as a major energy source in immune cells. During its metabolism, pyruvate is produced and then converted into acetyl-CoA in the mitochondria under aerobic conditions to generate energy efficiently. Under hypoxic conditions, pyruvate is converted to lactate in the cytoplasm (12). Lactate is traditionally regarded as a byproduct of anaerobic metabolism (13). However, increasing evidence reveals that lactate can also be produced under aerobic conditions and serves in various cellular roles, including as a major energy source, a key gluconeogenesis precursor, and a signaling molecule (14). For example, lactate activates the G protein-coupled receptor GPR81 on the cell surface, inducing biological processes such as energy metabolism, neuroprotection, and inflammation (15, 16). According to the lactate shuttle hypothesis, lactate can shuttle freely between cells via specific receptors, acting as a primary messenger for oxidation, gluconeogenesis, and cell signaling (17, 18). Additionally, lactate plays regulatory roles in both innate and adaptive immune cells (19) and induces significant changes in gene expression (20, 21).

Posttranslational modification (PTM) refers to the process by which proteins undergo covalent modifications, such as the addition of modification groups or cleavage at specific amino acid residues, leading to alterations in protein activity, subcellular localization, stability, and interactions. These changes play crucial roles in cellular signal transduction (22, 23). Recently, researchers identified a new PTM called lactylation, which is closely linked to abnormal glycolytic processes. Lactate accumulation during glycolysis induces lactylation, resulting in both histone and nonhistone modifications that play key roles in cellular functions and pathophysiological conditions through distinct regulatory pathways (15). Histone lactylation serves as an epigenetic mechanism to directly regulate chromatin gene expression, whereas nonhistone lactylation alters protein function by modifying structure (22). The discovery of this novel modification provides a new perspective on the intricate relationship between immune cell metabolic reprogramming and epigenetics.

Currently, the treatment of autoimmune diseases primarily involves substitution therapy (i.e., replacing enzymes or hormones that the body fails to produce), anti-inflammatory drugs, or immunosuppressive therapies. Although these approaches have shown some success in controlling disease progression, prolonged use of these treatments increases the risk of infections and cancer (24, 25). Consequently, safer and more effective therapeutic strategies are urgently needed. Lactate and lactylation are closely associated with the development of autoimmune diseases, and lactate production is intricately linked to immune cell metabolic reprogramming. Therefore, targeting lactate and lactylation may represent a novel therapeutic approach for treating autoimmune diseases. While several studies have investigated the link between lactylation and autoimmune diseases, the relationships between lactylation, immune cell metabolic reprogramming, and autoimmune diseases are still not fully understood. This article focuses on RA and SLE as case studies to explore the links between immune cell metabolic reprogramming, lactate, and lactylation in autoimmune diseases. This review also synthesizes the effects of lactate and lactylation on the physiological and pathological functions of immune cells, as well as their role in modulating gene expression via epigenetic modifications, which are implicated in the onset and progression of autoimmune disease. Furthermore, we discuss the therapeutic potential of targeting lactate production, lactylation, the combination of lactylation regulation with existing therapies, and the possibility of applying lactylation to personalized treatment in treating RA and SLE.

## 2 Lactylation: an emerging epigenetic modification

Epigenetic modifications, including various PTMs of histones and nonhistone proteins, can modulate gene expression patterns (26). These modifications are typically heritable and reversible, allowing for precise regulation of cell identity (27). In 2019, Zhang et al. identified lysine lactylation (Kla), a novel epigenetic modification. Kla is a prevalent histone lactylation where a lactyl group is covalently linked to lysine residues in proteins. This modification consequently influences protein function, stability, and interactions (22). Kla predominantly occurs on histones H3 and H4 and plays a key role in modifying chromatin structure and function, significantly impacting disease progression (15). Zhang’s team identified 28 Kla sites on core histones in human and mouse cells, demonstrating that histone Kla levels are regulated by glucose metabolism and changes in lactate concentration dynamics. They also showed that both exogenous and endogenous lactate directly induce histone Kla, whereas inhibiting key enzymes involved in lactate synthesis effectively suppressed this modification (20).

### 2.1 Key enzymes involved in lactylation

Lactate is converted to lactyl-CoA by CoA transferase (ALCT), with lactyl-CoA serving as the direct lactyl group donor for Kla (28). In 2019, Zhang et al. identified Megasphaera elsdenii ALCT as the most efficient catalyst for this conversion after screening five candidate enzymes. They also analyzed the stability of the three highest-performing ALCTs. These findings provide a deeper understanding of the mechanism of lactyl-CoA generation and offer new perspectives for the regulation of Kla (29). However, research on ALCT is still in its early stages, and detailed information is currently limited.

**Figure 1:**
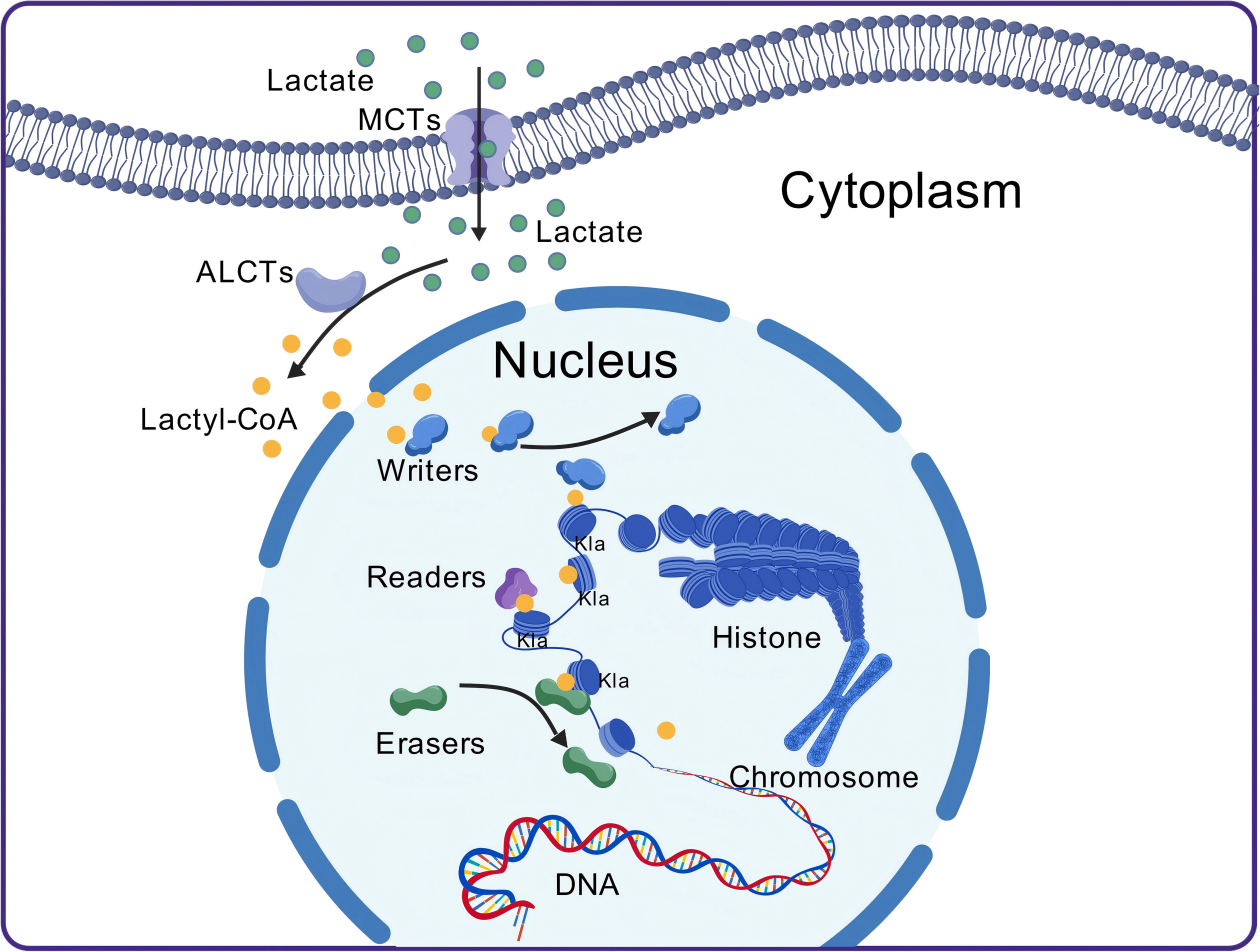
The process of lactylation. After lactate molecules enter the cell, they are converted into lactyl-CoA by CoA transferase. Lactyl-CoA then moves from the cytoplasm into the nucleus, where it is recognized and bound by the “writers.” The “writers” subsequently transfer the lactyl group onto histones. Histones that have undergone lactylation can then be recognized by the “readers,” which mediate their functional effects. The lactylation process is reversible, with “erasers” removing the lactyl group, thereby resulting in de-lactylation. MCT, monocarboxylate transporter; Kla, lysine lactylation; ALCT, CoA transferase.

Like acetylation, the occurrence of Kla depends on a series of enzymes responsible for adding lactyl groups to proteins, recognizing lactylation sites, and removing lactyl groups from proteins (30). Key enzymes associated with histone lactylation have been progressively identified, and these enzymes are generally considered epigenetic regulators, classified into three main categories: “writers,” which add lactyl groups to specific sites; “erasers,” which remove lactyl groups from proteins; and “readers,” which possess specific domains that can recognize lactylation sites (22, 31) (**Figure 1**).

#### 2.1.1 Writers

Studies have shown that lysine acetyltransferases (HATs) can mediate the transfer of the lactyl group from lactyl-CoA to proteins (32). CREB-binding protein (CBP) and E1A-binding protein P300 (p300) belong to the HAT family and exhibit high homology, similar amino acid sequences, and functions. Both act as coactivators for numerous transcription factors, and they are often collectively referred to as p300/CBP (33, 34). Previous studies have confirmed that p300/CBP can introduce Kla into proteins both inside and outside the nucleus, and moderate overexpression leads to elevated Kla levels (20). However, silencing p300/CBP expression in cells results in a significant decrease in Kla levels (35, 36). Additionally, research on H3K18la in monocytes (moDCs) revealed that general control nonderepressible 5 (GCN5), a classical HAT, also functions as a “writer” for Kla (37). p300 and GCN5 may work cooperatively during the Kla process (22). Despite evidence showing that both p300 and GCN5 can introduce Kla into proteins both inside and outside the nucleus, a systematic identification of all potential “writer” proteins is still needed to accurately quantify the contribution of each “writer” to Kla (30).

#### 2.1.2 Readers

Once the writers add the corresponding group to specific loci, the Kla can only affect recognition by other cellular proteins. These recognition proteins contain specialized domains capable of specifically identifying epigenetic marks and are therefore termed “readers” (38). Recent studies have indicated that the Brg1 protein accumulates at gene promoter regions associated with H3K18la during reprogramming, suggesting that Brg1 may act as a reader of H3K18la (39). However, further research is needed to determine whether the interaction between Brg1 and H3K18la is domain specific and whether Brg1 can also recognize Kla on other histones. Additionally, several specific protein domains have been identified and extensively studied in mammals. For example, proteins containing bromodomains can function as readers to regulate the transcriptional activity of target genes (40). Similarly, plant homologous structure finger protein 14 (PHF14) has been shown to act as a novel reader through an integrated PHD1/Znk/PHD2 (PZP) cassette (41). These discoveries provide new insights into the potential mechanisms underlying Kla, and future research should focus on verifying whether proteins containing bromodomains or PZP domains can function as Kla readers. While several potential readers have been identified in current studies, their precise role in the context of Kla remains unclear and warrants further investigation.

#### 2.1.3 Erasers

Lactylation is a reversible process, and histone deacetylases (HDACs) can remove the lactyl group, thus reversing Kla on histones (42). HDACs belong to the classical deacetylase family, which is divided into the zinc-dependent HDAC family (classes I, II, and IV) (43) and the NAD+-dependent HDAC SIRT family (class III HDACs, also known as sirtuins) (44). HDAC1–3 and SIRT1–3 have been shown to exhibit delactylase activity *in vitro*, with HDAC1–3 being the most stable and effective delactylases. Further experiments involving the overexpression and knockdown of HDAC1 and HDAC3 confirmed their delactylase activity in cells (42). Additionally, studies have identified SIRT2 as an effective *in vivo* histone Kla “eraser” capable of removing Kla from synthetic histone peptides, purified histones, and nucleosomes, as well as from multiple histone sites in neuroblastoma cells (45). It has also been reported that SIRT3 can eliminate lactylation at the H4K16la site, and its delactylase activity is more potent than that of other human sirtuins (46). Moreover, SIRT3 has nonhistone delactylase activity, removing lactylation from the K348la site of cyclin E2 (CCNE2) in hepatocellular carcinoma (HCC) cells (47). Therefore, both HDAC1–3 and SIRT1–3 function as Kla “erasers,” but the differences in their delactylase activities warrant further investigation.

In addition to histones, increasing numbers of nonhistone lactylation are being identified. Nonhistone lactylation affects a range of proteins beyond histones, including metabolic enzymes, transcriptional regulators, and signaling molecules. This modification affects the structure and function of chromatin by regulating metabolic pathways involving enzymes such as lactate dehydrogenase (LDH), pyruvate kinase (PK), and glycerol kinase (GK), these enzymes generate metabolites that serve as substrates for chromatin-modifying enzymes (e.g., transferases) (48–50), thereby influencing protein activity, stability, protein–protein interactions, and downstream binding capabilities. Compared to histone lactylation, nonhistone lactylation has a broader array of functions. In addition to regulating gene expression, nonhistone lactylation also acts as a signaling molecule, interacting with multiple intracellular signaling pathways such as the transforming growth factor-β (TGF-β/Smad2) and mitogen-activated protein kinase (MAPK) pathways to influence the progression of the disease (15, 51–53). Therefore, lactylation provides important insights into the mechanisms by which lactate regulates cellular metabolism and function. First, lactate promotes epigenetic changes via histone lactylation, thereby regulating the transcription of specific genes related to cellular functions and phenotypes. Second, lactate influences various proteins involved in carbohydrate, lipid, and amino acid metabolism through nonhistone lactylation, thus modulating metabolic processes. These two regulatory pathways are interconnected and exhibit complex interactions. Epigenetic regulation can affect cellular metabolism by altering the expression of metabolism-related genes, whereas metabolic adaptation can regulate epigenetic mechanisms by modulating the production of lactate or other metabolites (30).

### 2.2 Lactylation and acetylation

A key challenge in lactylation research is distinguishing its functional effects from acetylation due to their highly similar roles and shared lysine residues (20, 54). As discussed in this review, lactylation and acetylation share common enzymes during PTM processes. For instance, the acetyltransferase p300/CBP not only participates in lactylation but also catalyzes acetylation (55), while HDACs (histone deacetylases) exhibit dual activities in both deacetylation and delactylation. Therefore, there may exist a competition between lactylation and acetylation. Given that the modified state of proteins may act as “signaling switches” to regulate various biological processes within cells, this competitive relationship may play an important role in the broad regulation of cellular function and environmental adaptation (56–58). Findings from Zhang and team indicate genes marked by increased histone lactylation form a distinct group, as most do not exhibit parallel acetylation upregulation, thus implicating lactylation as a superior regulator of gene expression (20). Furthermore, lactylation and acetylation exhibit distinct temporal dynamics. Histone lactylation requires a significantly longer time (24 hours) to reach equilibrium compared to acetylation (6 hours). They occur at different time points or cellular states, thereby establishing a complex regulatory network (56).

## 3 The central role of immunometabolism in autoimmune diseases

### 3.1 Immune cell aerobic glycolysis

**Figure 2:**
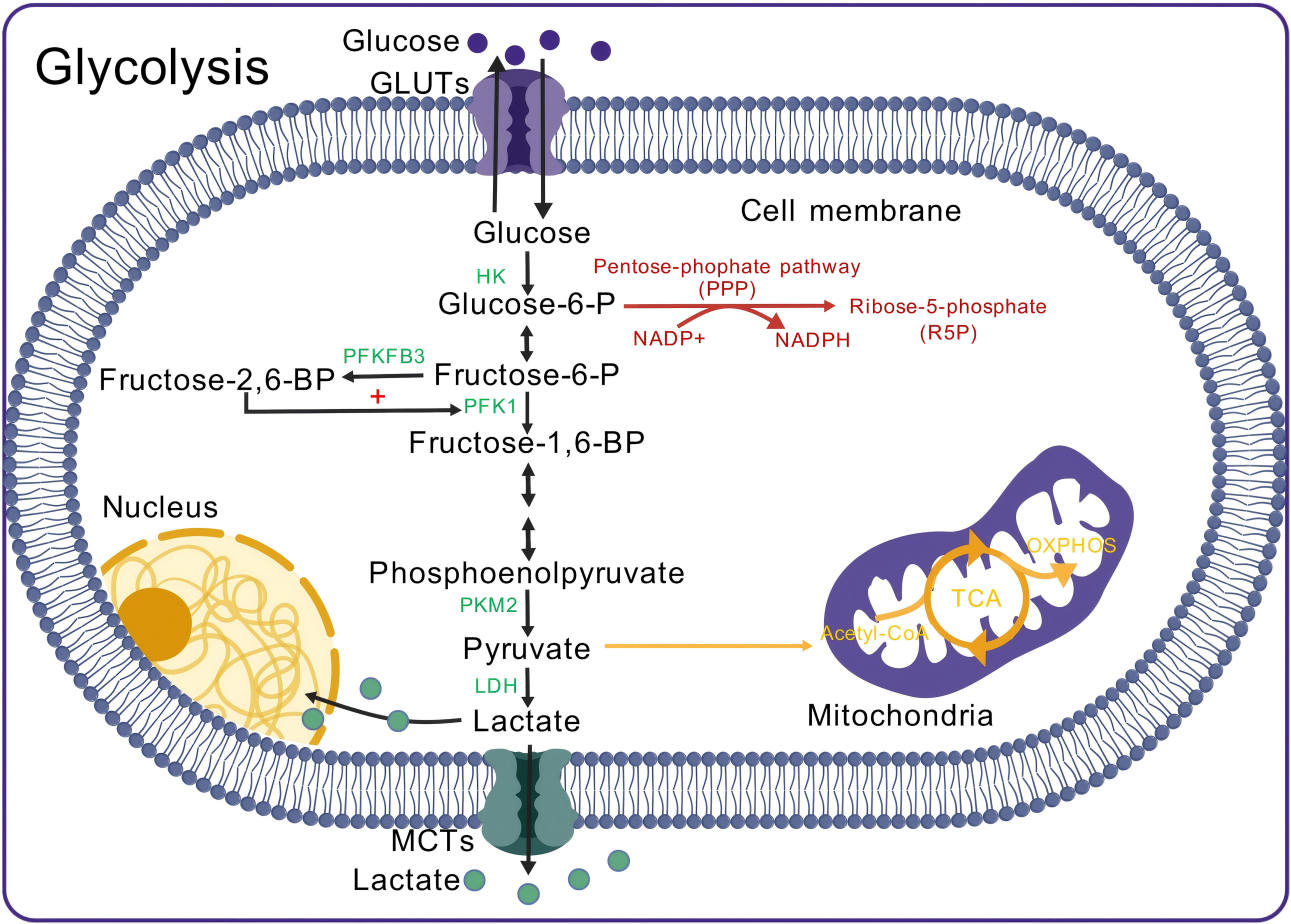
Aerobic glycolysis in immune cells. Glucose enters the cell and is phosphorylated by HK to form Glucose-6-P. The pentose phosphate pathway (PPP) operates alongside glycolysis, where Glucose-6-P serves as a substrate, generating NADPH, which is subsequently converted into R5P. R5P is essential for nucleotide synthesis, while NADPH serves as a reducing agent in biosynthesis and protects the cell from oxidative stress. In glycolysis, Glucose-6-P is converted into Fructose-6-P. PFKFB3 catalyzes the conversion of Fructose-6-P into Fructose-2,6-BP, which enhances PFK1 activity, promoting the conversion of Fructose-6-P to Fructose-1,6-BP. Fructose-1,6-BP undergoes several reactions to form phosphoenolpyruvate, which is then converted to pyruvate by PKM2. During aerobic glycolysis, 5% of pyruvate is converted to Acetyl-CoA, while 85% is converted to lactate by LDH. The generated lactate partly enters the nucleus, with the remainder transported outside the cell. GLUT, Glucose transporter; HK, Hexokinase; PFKFB3, 6-phosphofructo-2-kinase/fructose-2,6-bisphosphatase 3; PFK1, Phosphofructokinase 1; PKM2, Pyruvate kinase M2 isoform; LDH, Lactate dehydrogenase; MCT, Monocarboxylate transporter; Acetyl-CoA, Acetyl-CoA; TCA cycle, Tricarboxylic acid cycle; OXPHOS, Oxidative phosphorylation.

In normal immune cell energy metabolism, glucose enters the cell through glucose transporters (GLUTs) and is converted into pyruvate by various key enzymes. Pyruvate then enters the mitochondria, where it is converted into acetyl-CoA, which subsequently enters the tricarboxylic acid (TCA) cycle and produces ATP through oxidative phosphorylation (OXPHOS). Each glucose molecule in this pathway can ultimately produce 36 ATP molecules (59). However, when immune cells are activated and proliferate under certain conditions, they rely on glycolysis to meet rapid energy and biosynthesis demands, even under aerobic conditions. Pyruvate produced by glycolysis does not enter the TCA cycle or OXPHOS; instead, it is converted into lactate by LDH (12, 60, 61) (**Figure 2**). This process, known as the Warburg effect or aerobic glycolysis (62), results in continuous lactate production, leading to lactate accumulation and microenvironment acidification (63–65).

Aerobic glycolysis in immune cells is regulated by various signaling pathways. Mammalian target of rapamycin (mTOR) is a serine–threonine kinase that is part of the PI3K/AKT signaling pathway and plays a crucial role in regulating glycolysis (10). Upon activation by various kinases, mTOR activity is upregulated, promoting the expression of genes associated with glycolysis and inflammation (66). For example, mTOR enhances the activation of the hypoxia-inducible factor HIF-1α (67). HIF-1α upregulates LDHA expression, facilitating the reduction of pyruvate to lactate by LDHA and the lactate transporter MCT4 (68, 69). HIF-1α also activates glycolysis-related genes, such as GLUT1, HK, PKM2, and PFK, by binding to hypoxia response elements in gene promoters, thereby increasing their transcription and glycolytic activity to help immune cells meet their metabolic needs (70, 71).

According to the lactate shuttle hypothesis, lactate produced by glycolysis can be shuttled between cells and reutilized through specific receptors (72), such as MCTs (73, 74), G protein-coupled receptors (e.g., GPR81) (12) and hydroxycarboxylic acid receptor 1 (75). This intercellular lactate shuttle enables coordinated action, maximizing substrate utilization for energy production (72), but it may also lead to excessive lactate accumulation within cells, potentially leading to cellular damage (76).

### 3.2 Immune cell metabolic reprogramming

**Figure 3:**
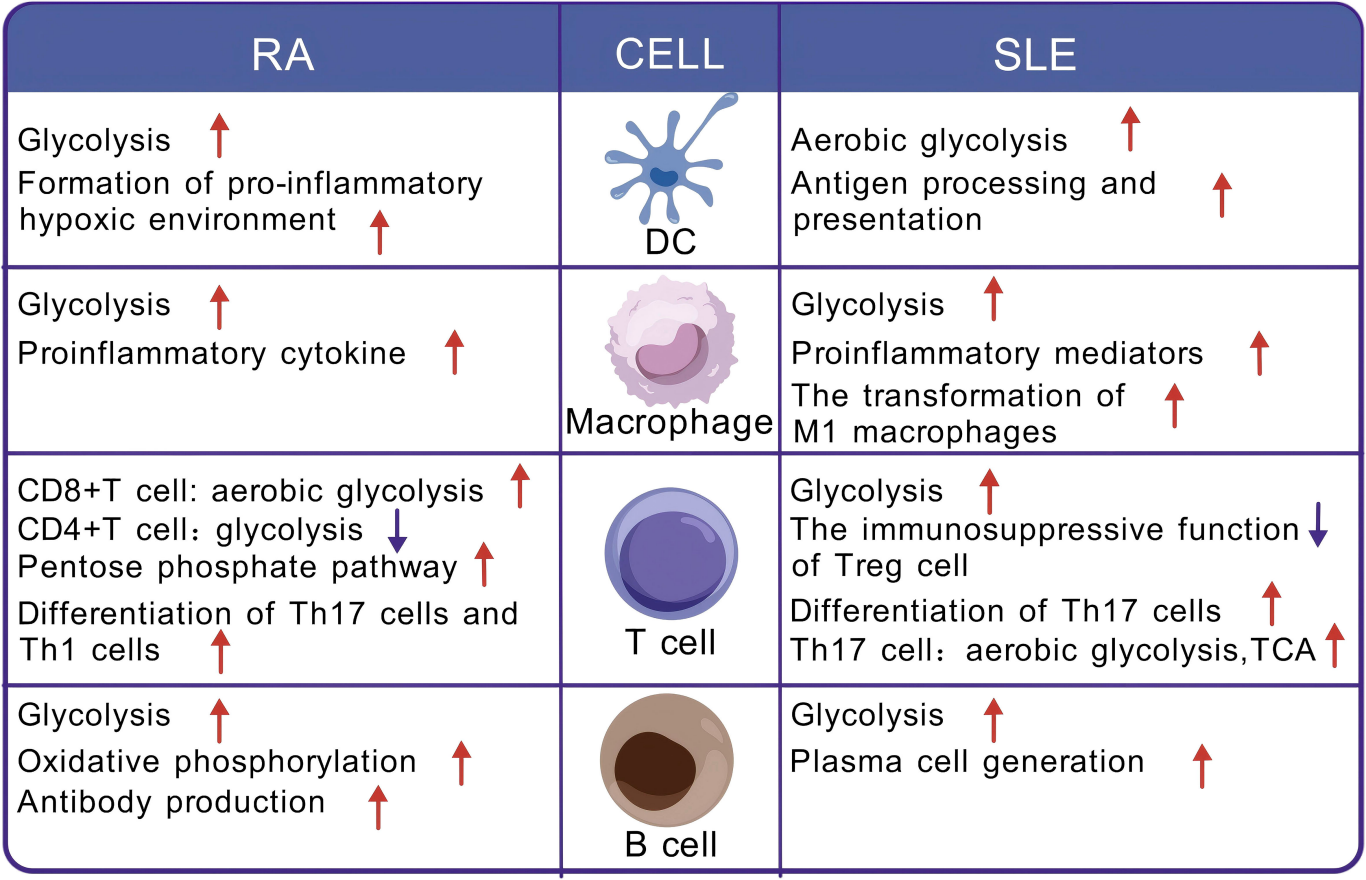
Metabolic reprogramming of immune cells in the context of rheumatoid arthritis and systemic lupus erythematosus. DC, dendritic cell; RA, rheumatoid arthritis; SLE, systemic lupus erythematosus; TCA, tricarboxylic acid cycle.

Immune cells undergo metabolic alterations in different states to meet the energy and biosynthetic needs required for proliferation, differentiation, and effector functions. This phenomenon is referred to as immune cell metabolic reprogramming (77–79). In autoimmune diseases, cells that participate in both innate and adaptive immune responses undergo metabolic reprogramming (**Figure 3**).

#### 3.2.1 Metabolic reprogramming of dendritic cells in autoimmune diseases

As the primary antigen-presenting cells in the peripheral immune system, dendritic cells (DCs) act as a link between innate and adaptive immune responses and are responsible for inducing lymphocyte activation and differentiation. In response to stimuli, the metabolism of dendritic cells gradually shifts from OXPHOS to glycolysis to meet their functional demands (10). Studies suggest that DC metabolic reprogramming is linked to autoimmune disease pathogenesis. In patients with RA, myeloid/conventional DCs (cDCs) are enriched in the synovium, and glycolysis is significantly increased (80). Furthermore, moDCs cultured ex vivo with synovial fluid exhibit a metabolic shift toward glycolysis. These metabolic changes in DCs may contribute to the establishment of an inflammatory hypoxic microenvironment in the RA synovium (80, 81). In patients with SLE, activated DCs undergo metabolic reprogramming, where aerobic glycolysis becomes the major metabolic pathway, providing energy and biosynthetic support for processing and presenting autoantigens (82, 83).

#### 3.2.2 Metabolic reprogramming of macrophages in autoimmune diseases

Macrophages play a critical role in innate immunity and exhibit different activation states depending on distinct metabolic patterns. Activated macrophages can be polarized into two phenotypes: M1 macrophages, which are typically associated with a proinflammatory phenotype and generate energy through aerobic glycolysis, and M2 macrophages, which are typically involved in immune regulation and tissue repair and rely on OXPHOS fuelled by fatty acid oxidation (FAO) (84, 85).The balance between macrophage phenotypes is critical for maintaining immune health, and dysregulation of this balance may contribute to autoimmune disease development. In RA patients, the balance between M1 and M2 macrophages is disrupted, exacerbating inflammation. In the synovial fluid of RA patients, 68% of macrophages are the proinflammatory M1 type (86). Studies have found an increased risk of coronary artery disease (CAD) in RA patients, with macrophages potentially exhibiting shared metabolic states in both diseases (87, 88). These cells enhance glycolysis by upregulating GLUT1, GLUT3, and key glycolytic enzymes (e.g., PKM2, PFKFB3, HK2) (87–89). M1 macrophages undergo metabolic reprogramming from OXPHOS to aerobic glycolysis to meet the energy demands of proliferation and activation while also releasing proinflammatory cytokines (90). In SLE patients, macrophages undergo a shift to an M1 phenotype dominated by glycolysis in response to IgG immune complex stimulation, and this metabolic reprogramming promotes the production of proinflammatory mediators, such as interleukin (IL)-1ß, further exacerbating the progression of SLE (91).

#### 3.2.3 Metabolic reprogramming of T cells in autoimmune diseases

As key cells that mediate host immune responses, T cells need to respond rapidly to foreign antigens and proliferate effectively within a short period to meet immune challenges. To meet the energy demands of this process, T cells undergo metabolic reprogramming (92). In the resting state, naïve T cells maintain low metabolic activity and produce ATP primarily through OXPHOS or FAO (93). However, when these cells are stimulated and differentiate into CD4+ and CD8+ T cells, their metabolism shifts to aerobic glycolysis. This process is accompanied by an increase in nutrient uptake, as well as the synthesis and accumulation of proteins, lipids, and nucleotides, promoting cell growth and proliferation (93). In contrast, regulatory T (Treg) cells and memory T cells rely primarily on OXPHOS derived from FAO for energy (94).

The pathogenesis of SLE is closely linked to the imbalance of Th subsets and Tregs, particularly the increased Th17:Treg ratio and the proliferation of follicular helper T (Tfh) cells (95, 96). In CD4^+^ T cells from SLE patients or lupus-prone mice, activation of the mTOR signaling pathway drives enhanced glycolysis, which promotes the differentiation of CD4^+^ T cells into pathogenic Th17 cells while compromising the immunosuppressive function of Treg cells (11, 97–100). The increased Th17 cells present elevated levels of aerobic glycolysis and TCA activity (99, 101, 102). Furthermore, in SLE patients or lupus-prone mice, Tfh cells display various metabolic dysregulation, leading to uncontrolled proliferation and providing excessive survival and differentiation signals to B cells in the germinal center (103–105). These findings suggest that metabolic reprogramming is closely associated with the differentiation and proliferation of autoimmune T cells in SLE. In patients with RA, CD8+ T cells undergo metabolic reprogramming through an mTOR/Myc/HIF-1α-dependent mechanism, shifting their metabolic profile toward aerobic glycolysis, which results in a proinflammatory and active phenotype (106, 107). In CD4+ T cells from RA patients, glycolysis is diminished, whereas the PPP is increased, leading to NADPH accumulation and reactive oxygen species (ROS) consumption, which drives T cells toward differentiation into highly aggressive and proinflammatory Th1 and Th17 cells (108, 109). This metabolic and differentiation change leads to the infiltration of synovial tissue by large numbers of proinflammatory Th cells, ultimately triggering joint damage (11, 110). Moreover, studies have shown that inhibiting glycolysis in RA animal models reduces the inflammatory function of CD4+ T cells, the terminal differentiation of B cells, and the severity of arthritis, further indicating that the progression of RA may be glycolysis dependent to some extent (111).

#### 3.2.4 Metabolic reprogramming of B cells in autoimmune diseases

B cells play a crucial role in autoimmune diseases, as they not only produce antibodies against self-antigens but also act as antigen-presenting cells, presenting self-antigens to self-reactive T cells (112). In adaptive immunity, mature follicular B cells initiate immune responses in germinal centers upon encountering antigens, generating memory B cells and plasma cells. During this process, B cells undergo metabolic reprogramming to obtain the energy and substrates required to support proliferation, differentiation, and function (113). Upon activation of the B-cell receptor (BCR), aerobic glycolysis is upregulated, increasing lactate production and initiating the germinal center response (114). Additionally, regulatory B (Breg) cells exert immune suppression in autoimmune diseases by secreting IL-10 (115), and their differentiation and proliferation depend primarily on glycolysis (116).

Immune metabolic dysregulation can lead to autoreactive B cells evading self-tolerance, thereby triggering the development of autoimmune diseases (117, 118). Under these conditions, the metabolic imbalance within autoreactive B cells contributes to the production of autoantibodies (119). In the B cells of SLE patients, the activation of the mTORC1 signaling pathway promotes glycolysis and drives plasmablast differentiation (119). T-bet+CD11c+ B cells, which accumulate in SLE patients, exhibit a hypermetabolic phenotype and rely on glycolysis for both differentiation and functional performance (120). In RA patients, B cells exhibit elevated glycolytic activity (121). Memory B cells accumulate in the synovium of RA patients and exhibit enhanced glycolytic activity, along with elevated T-cell costimulatory functions, proinflammatory cytokine production, and invasiveness (122). Additionally, plasma cells in RA patients accumulate in the synovium, where they increase glycolysis and OXPHO to support extensive antibody production (123, 124).

### 3.3 Glycolytic reprogramming and lactylation

Glycolytic reprogramming plays a critical role in maintaining the homeostasis of rapidly proliferating cells. The shift in cellular metabolism towards glycolysis not only provides rapid energy but also helps sustain the cellular redox balance. Additionally, glycolysis promotes lactate production, and an increase in lactate concentration can improve lactylation levels in a dose-dependent manner, establishing a positive correlation between lysine residue lactylation and the glycolysis rate (33, 125). Furthermore, as a PTM, lactylation can alter the structure and function of glycolysis-associated proteins, thereby influencing metabolic pathways (33). For example, lactylation is implicated in the activation and metabolic reprogramming of microglia, a process frequently observed in Alzheimer’s disease (AD). In AD brain tissue, histone H4K12la is enriched at the PKM2 promoter region, leading to elevated glycolysis and increased lactate production in microglia. This event initiates a positive feedback loop that perpetuates metabolic changes (126). However, recent studies have suggested that lactylation can also inhibit glycolytic reprogramming. In M1 macrophages, lactate promotes lactylation at the K26 site of PKM2, preventing its conformational shift from a tetramer to a dimer, which increases PKM2 activity and reduces its nuclear localization. As a result, lactylation interferes with glycolytic reprogramming in macrophages and supports their shift toward a repair phenotype (127). Thus, the impact of lactylation on glycolysis is context dependent and cell type specific, with effects varying on the basis of the cellular environment, metabolic demands, and pathological conditions.

## 4 Effects of lactate and lactylation on immune cells

### 4.1 Dual roles of lactate in macrophages

In the local microenvironment, lactate modulates immune responses by balancing the phenotypes of macrophages. Lactate can both suppress inflammation by promoting anti-inflammatory M2 macrophages and enhance the inflammatory response of proinflammatory M1 macrophages. M1 macrophages switch to aerobic glycolysis through metabolic reprogramming, resulting in lactate production, whereas M2 macrophages primarily utilize OXPHOS and FAO as their main metabolic pathways. These metabolic shifts are crucial in lactate-mediated immune regulation (20, 128).

Lactate exerts anti-inflammatory and reparative effects by promoting M2 macrophage polarization and inhibiting M1 macrophage functions. In M1 macrophages, lactate not only suppresses LPS-induced cytokine secretion (129) but also activates GPR81 to induce inhibitory signals that block Toll-like receptor (TLR)-4-mediated inflammasome assembly (130), thereby reducing the proinflammatory activity of M1 macrophages. Additionally, in tumor-associated macrophages, lactate can induce the expression of M2-associated genes through mechanisms such as MCT transport, HIF activation, inducible cyclic AMP early repressor, and epigenetic modifications (20, 131–134). These lactate-driven regulatory processes help reduce macrophage inflammation and regulate homeostasis. However, other studies suggest that lactate can promote inflammation under certain conditions. For example, lactate has been shown to increase the production of the inflammatory cytokine IL-23 in macrophages following LPS stimulation (135, 136). Additionally, lactate increases the ability of LPS-induced U937 cell lines and human monocyte-derived macrophages to secrete IL-6, matrix metalloproteinase-1, and IL-1ß and promotes proinflammatory NFκB activity through MCTs (137–139). These results demonstrate that the effects of lactate on inflammation in macrophages can vary, promoting or inhibiting inflammation depending on the context.

### 4.2 Lactylation and macrophage polarization

In innate immune cells, Kla is closely linked to macrophage polarization. During the differentiation of M0 macrophages into M1 or M2 phenotypes, Kla promotes macrophage plasticity by regulating the transcription of genes involved in polarization (18, 20). During M1 macrophage polarization, Kla positively influences the expression of M2-like genes—H3K18la promotes the transcription of repair genes associated with the M2 phenotype, such as arginase-1 (Arg1), driving the transition of macrophages from the proinflammatory M1 phenotype to the anti-inflammatory M2 phenotype (20). These findings suggest that histone Kla plays an important role in regulating the restoration of homeostasis during the resolution phase of immune inflammation.

In addition to histone lactylation, nonhistone lactylation also significantly contributes to macrophage polarization. PK, a critical enzyme in glycolysis, facilitates the conversion of phosphoenolpyruvate to pyruvate, representing the rate-limiting step in the glycolytic pathway (140). PKM2 is a PK isoform that is preferentially expressed in immune cells (141). The lactylation of PKM2 inhibits the inflammatory metabolic response in M1 macrophages and facilitates their transition from the M1 phenotype to the M2 phenotype. Exogenous lactate enhances this process by increasing PKM2 activity, which leads to increased expression of the M2 marker Arg1 in M2 macrophages and a reduction in elevated iNOS activity in M1 macrophages. Further research has shown that this effect can be reversed by inhibiting PKM2 activity, suggesting that lactylation promotes macrophage polarization toward the M2 phenotype by regulating PKM2, thereby accelerating tissue healing (127).

### 4.3 Dual roles of lactate in T cells

Lactate plays a complex dual role in the immune system by regulating immune suppression and inflammation through the modulation of T-cell metabolism, proliferation, and differentiation. The acidic environment created by lactate is crucial for maintaining the immunosuppressive function of Treg cells (142, 143). In the tumor microenvironment (TME), Tregs rely on the metabolic product lactate in the TME to maintain their suppressive function, thereby aiding tumors in immune escape (143). Lactate limits T-cell proliferation by altering the redox state of nicotinamide adenine dinucleotide (NAD^+^). In a lactate-rich environment, NAD^+^ is reduced to NADH by LDH, blocking NAD^+^-dependent enzymatic reactions associated with glyceraldehyde 3-phosphate dehydrogenase (GAPDH). This inhibits the glycolytic pathway involving GAPDH and reduces the production of 3-phosphoglycerate-derived serine, which is required for T-cell proliferation (144). Additionally, lactate accumulation inhibits the activity of Th cells while promoting the immunosuppressive function of Treg cells. Lactate increases Foxp3 expression by activating the ROS/IL-2 signaling pathway, leading to the conversion of Th17 cells to Treg cells (145). High expression of FOXP3 also reprograms Treg cell metabolism by inhibiting c-Myc and the glycolytic pathway, promoting OXPHOS, and enhancing NAD(+) reduction. This enables Treg cells to better adapt to low glucose and high lactate environments (142, 146). CD8^+^ T cells are critical for anti-tumor immunity, but in the TME, CD8^+^ T cells differentiate into exhausted Tex cells, and hypoxic environments upregulate MCT11 expression in these cells, thereby enhancing lactate uptake and metabolism in Tex cells, which exacerbates their dysfunction and subsequently facilitates tumor immune escape (147). However, another study shows that lactate can increase the stemness of CD8^+^ T cells and enhance anti-tumor immunity (148). These results suggest that the regulation of CD8^+^ T cell function by lactate may exhibit high complexity and environmental dependence.

Although lactate enhances the immunosuppressive effects mediated by Treg cells, studies have also shown that lactate can drive the differentiation of naïve CD4+ T cells into inflammatory Th17 cells. Elevated lactate levels in the immune microenvironment induce the expression of the lactate transporter SLC5A12 in CD4+ T cells, driving lactate influx. In CD4+ T cells, the influx of lactate enhances IL-17 production in a manner dependent on retinoic acid receptor-related orphan receptor gamma (RORγt) via the nuclear PKM2/STAT3 signaling pathway, simultaneously inducing Th17 cell polarization and further exacerbating the inflammatory response (149). The reviewed studies predominantly rely on mouse models, and the effects of lactate on human T cells require further experimental exploration.

Additionally, glycolysis in tumor cells and the resulting elevated lactate concentrations promote the differentiation of MDSCs (150). MDSCs are immunosuppressive cells that perform a wide range of immune-suppressive functions by modulating T-cell responses (146, 151).

### 4.4 Role of lactylation in T-cell function

Lactylation regulates the epigenetic profile of T cells, affecting the equilibrium between proinflammatory Th17 cells and immunosuppressive Treg cells. Lactate can reprogram Th17 cells into Treg cells with immunosuppressive functions via Kla (145). After lactate treatment, the level of histone H3K18la in Th17 cells increases, significantly decreasing IL-17A production, while the expression of the Treg signature transcription factor Foxp3 is significantly upregulated. However, other studies have shown that the lactylation of specific proteins can enhance Th17 cell differentiation. Lactylation of Ikaros family zinc finger 1 at the K164 site can regulate its binding to the promoters of Th17-related genes, differentially regulating the expression of these genes and ultimately promoting the differentiation of Th17 cells (152). These results suggest that the effect of lactylation on T cells depends on the type of protein that undergoes lactylation, which is variable.

In addition, lactylation can influence T-cell differentiation through the TGF-β signaling pathway. In naïve T lymphocytes, TGF-β signaling promotes the differentiation of Treg cells. In an IL-6-deficient environment, lactylation of MOESIN at position K72 activates the TGF-β pathway, enhancing the binding of MOESIN to TGF-β receptor I and promoting downstream SMAD3 signaling, which in turn induces Treg differentiation (53). However, in the presence of IL-6, TGF-β combines with IL-6 to increase Th17 differentiation (153). IL-6 is known to play a key role in various autoimmune diseases, with significantly elevated levels in the synovial fluid of RA patients (154, 155). Nevertheless, the effect of lactylation on T-cell differentiation via TGF-β signaling in IL-6-related autoimmune diseases remains to be further investigated.

Recent studies have shown that histone lactylation plays a crucial role in regulating CD8 T-cell metabolism and the transcription of key genes involved in their function. CD8+ T-cell activation promotes lactate production, which subsequently increases the levels of H3K18la and H3K9la. These elevated levels of H3K18la and H3K9la serve as transcriptional initiation signals for genes that regulate CD8 T-cell function. Targeting metabolic and epigenetic pathways to inhibit H3K18la and H3K9la significantly reduces the expression of effector genes in T cells and suppresses CD8 T-cell-mediated cytotoxicity (156). These findings suggest that H3K18la and H3K9la may enhance the effector function of CD8+ T cells.

### 4.5 Lactate and the immune microenvironment

The dual role of lactate is closely tied to the immune microenvironment. Lactate is a weak acid that easily dissociates into lactate ions and H+, and its accumulation in the extracellular space leads to immune microenvironment acidification and acidosis, affecting immune cell function and the strength of immune responses (157–159). Additionally, soluble mediators and cytokines in the immune microenvironment can modulate the impact of lactate on immune cell differentiation. When monocytes differentiate in the presence of lactate and granulocyte–macrophage colony–stimulating factor (GM-CSF) or in conditioned medium rich in lactate and other factors from adenocarcinoma, there is a simultaneous increase in the production of both M1 and M2 mediators (160, 161). Further experiments have revealed that GM-CSF and lactate jointly drive the production and consumption of IL-6-dependent macrophage colony-stimulating factor which promotes the formation of an inflammatory feedforward loop (139, 160).

## 5 Lactate and lactylation in specific autoimmune diseases

### 5.1 Rheumatoid arthritis

RA is a chronic, inflammatory, systemic autoimmune disorder (162). In the early stages of RA, the body’s immune tolerance mechanisms are disrupted, leading to immune system dysregulation. Persistently proliferating CD4+ T cells produce large amounts of proinflammatory cytokines, chemokines, and angiogenic factors, promoting the infiltration, proliferation, and differentiation of various immune cells, such as synovial fibroblasts, macrophages, and dendritic cells (163). As the disease progresses, osteoclasts become more active, promoting bone resorption and leading to cartilage destruction and bone erosion (164). Lactate plays a critical role in catalysing the development of RA. Studies have shown that in the synovial fluid of RA patient, glucose levels are lower, and lactate levels are significantly elevated (165). In the inflammatory microenvironment, immune cells undergo aerobic glycolysis, which regenerates NAD+, supporting the proinflammatory phenotype (166). During this process, lactate accumulates and is transported to the extracellular environment, where it enters CD4+ T cells, macrophages, dendritic cells, and osteoclasts through lactate transporters along the concentration gradient (167). This results in the differentiation and activation of these cells, altering their function and ultimately exacerbating RA progression (168, 169).

Lactate promotes the differentiation of T cells into Th17 cells in RA patients. In the synovial fluid of RA patients, lactate accumulation enhances the expression of the lactate transporter SLC5A12 on CD4+ T cells, which mediates lactate uptake and alters the effector phenotype of these cells (149). The lactate taken up by these cells stimulates fatty acid synthesis, resulting in increased palmitic acid production. Palmitic acid then triggers the phosphorylation of signal transducer and activator of transcription 3 (STAT3), which activates the expression of the downstream RORγt gene, promoting IL-17 production and the differentiation of Th17 cells (170). Th17 cells play a potent proinflammatory role in RA, exacerbating cartilage destruction and bone erosion, which accelerates disease progression (171). Furthermore, lactate enters both CD4+ and CD8+ T cells via SLC5A12 and MCT1, respectively, inducing a “stop migration” signal that causes these cells to accumulate at the site of inflammation (149, 168). Lactate is also taken up by B cells through lactate transporters, endowing them with elevated glycolytic activity and increased inflammatory characteristics, which promote the continuous infiltration of B cells in the RA synovium (121, 172). Analysis of peripheral blood mononuclear macrophage phenotypes in RA patients revealed upregulation of the M1 markers CD80 and CD86, whereas the M2 marker CD163 was downregulated (173). In RA patients, multiple signaling pathways, including the Notch, JAK/STAT, NF-κb and MAPK pathways, participate in regulating the polarization of macrophages to the M1 phenotype (174). Consequently, lactate may facilitate the transformation of macrophages into the M1 phenotype through multiple mechanisms. Additionally, lactate stimulates the formation of osteoclasts and increases their activity. A study on breast cancer has found that osteoclasts take up lactate via MCT1 and utilize it for OXPHOS, which accelerates bone resorption (175). However, further experimental validation of this mechanism in RA is warranted. Fibroblast-like synoviocytes (FLSs) represent the predominant synoviocyte type. In RA patients, FLSs undergo metabolic reprogramming from OXPHOS to glycolysis, resulting in increased proliferation, resistance to apoptosis, and invasive properties (176). These cells secrete inflammatory and proangiogenic factors to sustain joint inflammation and promote angiogenesis. Moreover, they produce matrix metalloproteinases (MMPs) and receptor activator of nuclear factor κB ligand (RANKL), contributing to cartilage and bone destruction (177, 178). FLSs also absorb lactate from the microenvironment through MCT1, which not only promotes glycolysis, migration, and invasion but also increases IL-6 expression (140, 179, 180). The latest research indicates that lactylation in plasma cells may promote the formation of an inflammatory environment in RA. The plasma cells of RA patients exhibit increased levels of lactylation and enhanced OXPHOS and glycolysis, which are crucial for meeting the high energy demands of the autoantibody production process. The significant upregulation of lactylation in plasma cells suggests that there may be a novel regulatory mechanism in RA that affects the autoantibody production and immune activation (123).

In RA patients, metabolically active immune cells such as fibroblast-like synoviocytes, macrophages, B cells, and T cells undergo metabolic reprogramming in response to environmental factors such as hypoxia, nutrient deprivation, and inflammatory stimuli. They switch to glycolysis as the primary metabolic pathway to rapidly acquire energy and synthesize precursors. This shift supports the pathological proliferation of these immune cells, while the lactate produced during glycolysis regulates their metabolism, development, and function through signaling pathways and lactylation, further driving the progression of the disease.

### 5.2 Systemic lupus erythematosus

SLE is a chronic, multifactorial autoimmune disorder. In SLE, the immune system becomes aberrantly activated, producing IgG autoantibodies that target self-antigens. This leads to persistent inflammatory responses and tissue damage, establishing a vicious cycle (181).

**Figure 4:**
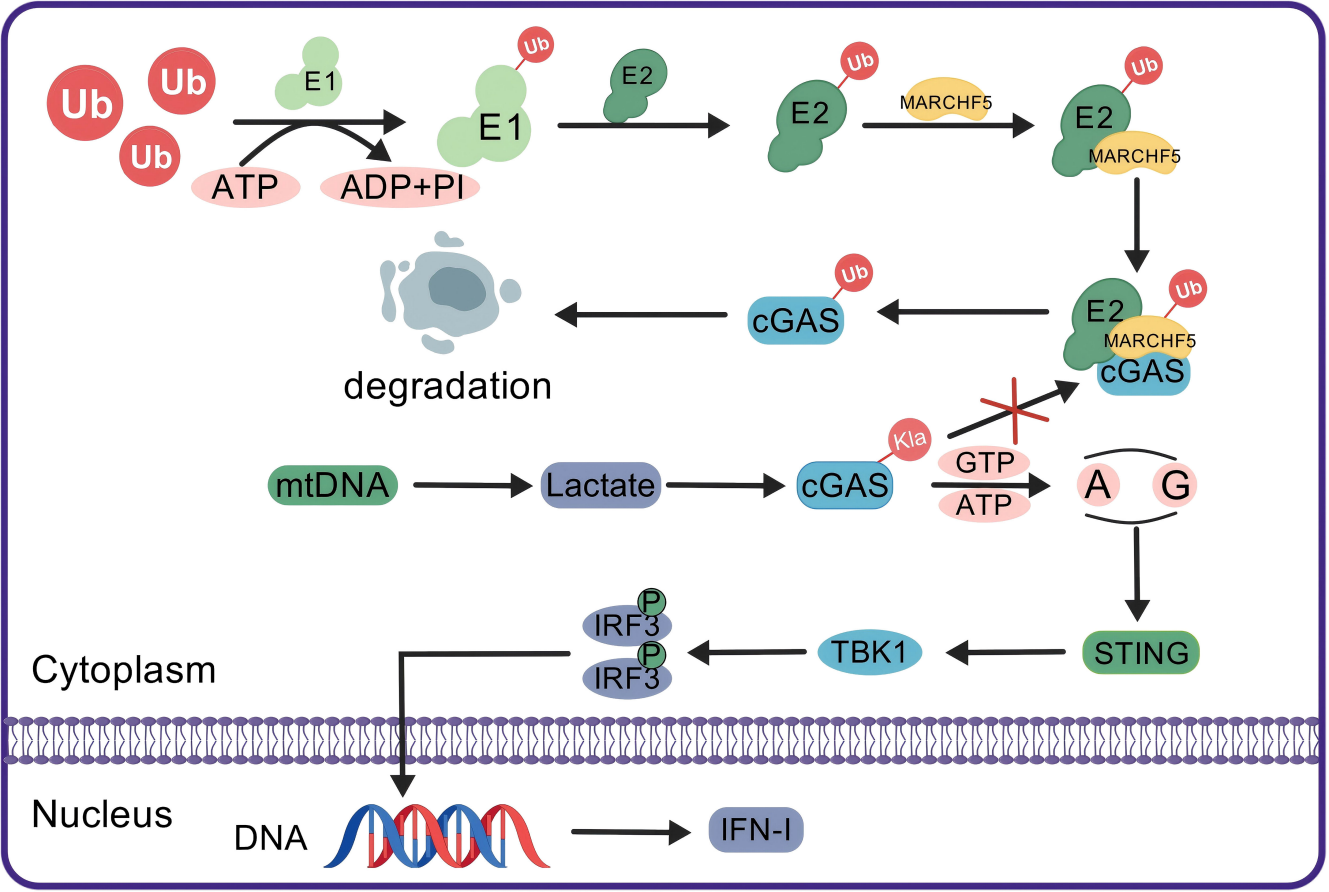
Lactylation enhances IFN-I production by blocking the ubiquitination and degradation of cGAS. In the normal ubiquitin-proteasome system, the Ub molecule is activated by E1 through ATP hydrolysis and then transferred to E2. The E2-Ub complex marks cGAS for degradation via MARCHF5, an E3 ubiquitin ligase. In patients with SLE, mtDNA stimulation enhances cellular glycolysis, resulting in increased lactate production. The accumulation of lactate leads to the lactylation of cGAS, which blocks its ubiquitination. The undisturbed cGAS activates downstream STING signaling, which in turn activates TBK1. TBK1 then phosphorylates IRF3, which translocates to the nucleus to promote DNA transcription and production of IFN-I, thereby enhancing the immune response. Ub, ubiquitin; E1, ubiquitin-activating enzyme; E2, ubiquitin-conjugating enzyme; MARCHF5, an E3 ubiquitin ligase; cGAS, cyclic GMP-AMP synthase; mtDNA, mitochondrial DNA; Kla, histone lysine lactylation; STING, stimulator of interferon genes; TBK1, TANK-binding kinase 1; IRF3, interferon regulatory factor 3; IFN-I, type I interferon.

In SLE, abnormal nucleic acid signaling pathways lead to an increase in type I interferon (IFN-I) production, thereby increasing the production of IgG autoantibodies (182, 183). As an immune adjuvant, IFN-I facilitates the activation of the systemic immune system, particularly benefiting B-cell differentiation and the production of proinflammatory chemokines, thereby playing a pivotal role in the sustained autoimmune responses observed in SLE patients (184). Recent research has demonstrated that cytosolic mitochondrial DNA (mtDNA) in SLE can induce glycolytic reprogramming in macrophages, subsequently promoting aerobic glycolysis and leading to significant lactate production. The accumulation of lactate promotes the lactylation of cyclic GMP-AMP synthase (cGAS), thereby inhibiting its ubiquitination and degradation, and ultimately stimulates the generation of IFN-I by amplifying the cGAS/STING signaling pathway (**Figure 4**). These results demonstrate that lactate and lactylation promote the progression of SLE (185).

Additionally, lactate may influence organ damage associated with SLE. Lupus nephritis (LN), a common form of glomerulonephritis, is one of the most important early organ manifestations of SLE (178). Recent studies have shown that serum-derived lactate-related biomarkers, including COQ2 (coenzyme Q2), COQ4, and NADH: Ubiquinone Oxidoreductase Core Subunit V1 (NDUFV1), could contribute to the development of LN through mechanisms such as “antigen processing and presentation” and the “NOD-like receptor signaling pathway” (186).

## 6 Lactylation as a therapeutic target

### 6.1 Development of lactylation inhibitors

Lactylation, a newly recognized epigenetic modification, has been identified as a marker of the glycolytic switch, suggesting a novel direction and potential therapeutic targets for investigating the role of lactate in autoimmune diseases (146). Inhibiting enzymes related to lactylation can reduce lactylation and suppress the expression of proinflammatory genes. The use of small molecules or gene therapies to regulate lactylation-related enzymes, such as p300/CBP and HDAC, may provide effective therapeutic strategies (30). Although the specific reader proteins for Kla have not been identified, blocking the recognition of Kla by site-specific readers could represent another potential treatment approach for autoimmune diseases (22). Recent research has shown that evodiamine can inhibit H3K18la and serve as a direct modulator of lactylation (187), although its potential for treating autoimmune diseases through lactylation regulation remains to be fully explored. Additionally, while targeting ALCT, a key enzyme in the conversion of lactate to lactyl-CoA (29), has been proposed as a therapeutic strategy, the lack of a comprehensive understanding of its complete activity and mechanisms in mammals means that the use of ALCT inhibitors for precise treatment remains a distant possibility.

### 6.2 Regulation of lactate production and secretion

Research has shown that lactylation is highly sensitive to lactate levels (20). Therefore, targeting the glycolytic pathway, which generates lactate, may provide a promising strategy for lactylation-based therapies (15). Glycolysis relies on the action of key enzymes, and developing inhibitors of these enzymes could modulate lactylation in autoimmune diseases (188, 189). For example, 2-deoxy-D-glucose (2-DG) and 3-bromopyruvate (3-BP) inhibit HK, the first rate-limiting enzyme in glycolysis (190, 191). Preventive treatment with these inhibitors suppresses glycolysis, thereby reducing lactate production and indirectly modulating lactylation levels. These metabolic shifts may attenuate the activation of both adaptive and innate immune cells and reduce the production of pathogenic autoantibodies subsequently (82, 111, 192, 193). Notably, glycolytic inhibitors like 2-DG act on multiple metabolic pathways (e.g., glycolysis, PPP, TCA cycle) (194, 195) rather than solely targeting lactate production. While reduced lactylation may occur, direct proof linking this to immune regulation is lacking. Thus, avoiding overinterpretation of lactylation’s role is essential until mechanistic studies confirm causality.

Certain bioactive compounds derived from natural products have shown potential in treating autoimmune diseases by modulating lactate production. For example, FX11 (3-dihydroxy-6-methyl-7-(phenylmethyl)-4-propylnaphthalene-1-carboxylic acid) reduces lactate production by competitively binding to the active site of LDH (196). Curcumin limits glucose uptake by directly binding to GLUT1, a glucose transporter, thus inhibiting glycolysis and reducing lactate production (197, 198). Moreover, developing drugs that inhibit lactate transporters, such as MCT4 and SLC5A12, could reduce lactate secretion and lower its concentration in the extracellular space, thereby alleviating disease severity (168). Furthermore, 4′-diisothiocyano-2,2′-stilbenedisulphonate (DIDS)-mediated MCT1 inhibition not only blocks lactate efflux (199) but also impairs MCT1-dependent lactate uptake by CD4^+^ T cells, CD8^+^ T cells, and FLS in RA, thereby disrupting pro-inflammatory metabolic signaling and alleviating joint inflammation (**Table 1**).

**Table 1:** Glycolysis inhibitors and their mechanism of action.

Inhibitor	Target	Mechanism of action	References
2-DG	HK	2-DG engages in competition with glucose for binding transmembrane GLUTs, facilitating its cellular entry. Subsequently, HK catalyzes the phosphorylation of 2-DG, generating 2-deoxy-D-glucose-6-phosphate (2-DG-6P), which becomes retained within the cells. Owing to the inability of 2-DG-6P to undergo further metabolism by phosphofructoisomerase, the glycolysis pathway experiences inhibition.	(200–202)
3-BP	HK2	HK catalyzes the first step of glycolysis. The 3-BP irreversibly alkylates HK2 and thereby inhibiting glycolysis.	(192, 203)
FX11	LDHA	It inhibits the glycolytic process, wherein LDHA catalyzes the conversion of pyruvate to lactate, by competitively binding to LDHA, thereby displacing NADH and disrupting the enzymatic reaction.	(196)
Curcumin	GLUT1	Curcumin is capable of binding to GLUT1 and directly inhibiting its activity, thereby suppressing glycolysis by inhibiting glucose transport.	(198)
Phloretin	MCT4	It forms an aromatic-H bond with tyrosine 36 in MCT4, inhibiting the function of MCT4, thereby affecting the extracellular secretion of lactate produced via glycolysis.	(197, 204)
α-CHCA (α-Cyano-4-hydroxycinnamate)	MCT4	Targeting glycosylation of extracellular matrix metalloproteinase inducer (EMMPRIN) impedes its interaction with MCT4, preventing MCT4 from localizing at the plasma membrane. This interruption curbs the secretion and transport of Lactate.	(197, 205)
DIDS	MCT1	DIDS irreversibly binds to lysine residues on MCT1, thereby impairing its ability to transport lactate and inhibiting the secretion of lactate produced via glycolysis.	(199)

Although inhibiting glycolysis holds therapeutic promise, potential off-target effects must be acknowledged. Glycolysis is a fundamental metabolic pathway in cells with high energy demands, such as rapidly dividing immune cells (e.g. activated T cells and intestinal epithelial cells) (206–208). Inhibition of glycolysis may disrupt energy homeostasis in these normal cells, leading to unintended consequences like immune suppression or gastrointestinal toxicity. Therefore, further preclinical research is essential to systematically evaluate the safety profile and long-term effects of glycolysis-targeted therapies.

### 6.3 Combination therapy with existing treatment strategies

In autoimmune diseases, autoantigens and endogenous stimuli continuously activate the immune system, resulting in persistent inflammation (209). In the tissue microenvironment of chronic inflammation, aerobic glycolysis increases, which may lead to elevated lactate levels (210). Studies have shown that lactate, as a regulatory factor, can either suppress or enhance inflammatory responses and is therefore crucial for the maintenance of inflammatory homeostasis (16). Additionally, lactylation is closely linked to inflammation and plays a significant role in modulating inflammatory responses. Therefore, combining lactylation-modulating drugs with existing anti-inflammatory treatments could further improve the management of autoimmune diseases. For example, combining lactylation-modulating drugs with anti-inflammatory agents, such as IL-17 inhibitors, may more effectively control chronic inflammation in diseases such as RA. Research into such combination therapies is promising for enhancing treatment precision and minimizing side effects.

### 6.4 Application of personalized treatment strategies

As personalized medicine continues to develop, lactylation holds promise as a scientific basis for more precise treatment strategies. In patients who are receiving targeted metabolic therapy for autoimmune diseases, there is often considerable variability in drug tolerance and efficacy. By exploring these individual differences in lactylation levels and their potential in personalized medicine, treatment plans can be tailored to each patient, providing a strong foundation for the advancement of precision medicine (211). However, the application of lactylation in personalized medicine still requires substantial clinical research and validation to ensure its safety and practicality (212).

## 7 Conclusion and prospects

Lactylation, a key PTM, regulates protein structure and function and, through epigenetic modifications, influences gene expression, thus playing a role in various physiological and pathological processes (33). By modulating lactate production and lactylation, scientists have begun to identify the critical roles of lactate in immune cell function, inflammatory responses, and metabolic reprogramming. Lactate impacts immune cell epigenetic changes and metabolic processes through both histone and nonhistone lactylation, which are closely linked to the pathogenesis of autoimmune diseases. In RA patients, for example, lactate accumulates in the inflammatory tissue microenvironment, providing an unregulated signal for glycolysis that induces inflammatory cytokine production and mediates abnormal immune cell development and differentiation (168). In SLE patients, lactylation enhances IFN production by inhibiting the ubiquitination of cGAS, thus promoting the generation of autoantibodies. The metabolic reprogramming of immune cells in autoimmune diseases is associated with lactylation levels: lactate produced during glycolytic reprogramming influences lactylation, while lactylation regulates glycolysis by modulating the transcription and functional structure of key enzymes. Consequently, the abnormal metabolic reprogramming of immune cells may contribute to disease progression. Lactate and lactylation play dual roles in the immune system, with the complex actions of lactate arising not only from its acidic properties but also from its synergistic effects with other cytokines and solutes in the immune microenvironment. This synergy plays a vital role in regulating immune cell differentiation and modulating immune response intensity. Future research should further explore how lactate and the immune microenvironment specifically control immune cell differentiation and immune response regulation.

As a link between metabolism and epigenetics, lactylation offers novel therapeutic insights for autoimmune diseases. Mechanistically, innovations in therapies targeting enzymes involved in the lactylation process and the regulation of lactate production, along with integrative approaches with existing treatment protocols, may yield breakthroughs in managing autoimmune conditions such as RA, SLE, and multiple sclerosis. Elucidating the specific mechanisms of lactylation and developing targeted therapeutic drugs could pave the way for precision medicine and personalized treatment of autoimmune diseases. Although this field is still nascent, its potential for application is immensely promising.
